# Limited handwashing facility and associated factors in sub-Saharan Africa: pooled prevalence and multilevel analysis of 29 sub-Saharan Africa countries from demographic health survey data

**DOI:** 10.1186/s12889-022-14390-4

**Published:** 2022-10-27

**Authors:** Mastewal Endalew, Daniel Gashaneh Belay, Nuhamin Tesfa Tsega, Fantu Mamo Aragaw, Moges Gashaw, Melaku Hunie Asratie

**Affiliations:** 1 Department of Environmental and Occupational Health and Safety, Institute of Public Health, College of Medicine and Health Sciences, University of Gondar, Gondar, Ethiopia; 2 Department of Human Anatomy, College of Medicine and Health Sciences, University of Gondar, Gondar, Ethiopia; 3 Department of Epidemiology and Biostatistics, Institute of Public Health, College of Medicine and Health Sciences, University of Gondar, Gondar, Ethiopia; 4 Department of Women’s and Family Health, School of Midwifery, College of Medicine and Health Sciences, University of Gondar, Gondar, Ethiopia; 5 Department of Physiotherapy, School of Medicine, College of Medicine and Health Science, University of Gondar, Gondar, Ethiopia

**Keywords:** Limited handwashing facility, DHS, Sub-Saharan Africa, Multilevel regression

## Abstract

**Introduction:**

Handwashing is fundamentally an inexpensive means of reducing the spread of communicable diseases. In developing countries, many people die due to infectious diseases that could be prevented by proper hand hygiene. The recent coronavirus (COVID-19) pandemic is a threat to people who are living in resource-limited countries including sub-Saharan Africa (SSA). Effective hand hygiene requires sufficient water from reliable sources, preferably accessible on premises, and access to handwashing facility (water and or soap) that enable hygiene behaviors. Therefore, this study aims to determine the prevalence of limited handwashing facility and its associated factors in sub-Saharan Africa.

**Methods:**

Data from the Demographic and Health Surveys (DHS) were used, which have been conducted in 29 sub-Saharan African countries since January 1, 2010. A two-stage stratified random cluster sampling strategy was used to collect the data. This study comprised a total of 237,983 weighted samples. The mixed effect logistic regression model with a cluster-level random intercept was fitted. Meta-analysis and sub-group analysis were performed to establish the pooled prevalence.

**Results:**

The pooled prevalence of limited handwashing facility was found to be 66.16% (95% CI; 59.67%—72.65%). Based on the final model, household head with age group between 35 and 60 [AOR = 0.89, 95% CI; 0.86—0.91], households with mobile type of hand washing facility [AOR = 1.73, 95% CI; 1.70—1.77], unimproved sanitation facility [AOR = 1.58, 95% CI; 1.55—1.62], water access more than 30 min round trip [AOR = 1.16, 95% CI; 1.13—1.19], urban residential area [AOR = 2.08, 95% CI; 2.04—2.13], low media exposure [AOR = 1.47, 95% CI; 1.31—1.66], low educational level [AOR = 1.30, 95% CI; 1.14—1.48], low income level [AOR = 2.41, 95% CI; 2.33—2.49] as well as lower middle-income level [AOR = 2.10, 95% CI; 2.14—2.17] and households who had more than three children [AOR = 1.25, 95% CI; 1.20—1.31] were associated with having limited handwashing facility.

**Conclusion and recommendation:**

The pooled coverage of limited handwashing facility was high in sub-Saharan Africa. Raising awareness of the community and promoting access to handwashing materials particularly in poorer and rural areas will reduce its coverage.

**Supplementary Information:**

The online version contains supplementary material available at 10.1186/s12889-022-14390-4.

## Introduction

Handwashing is a fundamental principle and a low-cost method of minimizing communicable diseases transmission [1, 2]. Hand hygiene is seen as a critical intervention strategy for pandemic public health threats. According to evidence from systematic reviews and clinical interventions, handwashing with soap can reduce the risk of diarrheal infection by 30% to 47% [3–5] and the risk of acute respiratory infections by 16% [6]. Pervious meta-analysis finding showed that, an increase in hand hygiene resulted reductions in gastrointestinal and respiratory illness [7]. Based on a joint report of the World Health Organization (WHO) and United Nations Children's Fund (UNICEF), two out of every five people in the world have limited handwashing facility (lack water plus detergents), with a high percentage of these people coming from the least developed countries [8]. As a result, many people died due to infectious diseases that could have been prevented by proper hand hygiene. Study done in forty-four countries indicated that 35.5% of the population are living with a limited handwashing facility (lacks soap and/or water) [9]. In sub-Saharan Africa, the coverage of limited handwashing facility is 85% [8, 10]. Evidence from Ethiopia Demographic Health Survey (EDHS) indicated that, 53.04% of households have limited handwashing facility (handwashing places available but neither water nor detergent observed on-premises [11].

Based on WHO report, over 270 million confirmed cases of coronavirus and more than 5 million confirmed deaths were registered globally [12]. The recent coronavirus (COVID-19) pandemic is a threat to people who are living in resource-limited countries including sub-Saharan Africa (SSA). Although it can be prevented by effective handwashing practices [8, 9]. According to WHO, unsafe water, inadequate sanitation, or insufficient hand hygiene causes 88% of diarrhea cases worldwide. This diarrheal disease kills 1.5 million people per year, the vast majority of whom are children [13]. Effective hand hygiene requires sufficient water from reliable sources, preferably onsite, and accessible handwashing facilities that facilitate hygiene behaviors [14]. However, access to water and soap is deficient especially in low and middle—income countries. Handwashing with soap and water is the most effective means of preventing COVID-19 infection, both mechanically and by altering viral integrity [15]. Soap molecules break the outer lipid membrane of the microbe, causing viral fragments to run away with water [15]. Handwashing is a routine exercise for people in rich countries, but it is not for people in developing [16]. Water and soap are still in short supply in rural areas and urban slums [16]. In the absence of soap and water, hand sanitizers are used to clean hands. However, the potential problematic health outcomes and flammability of alcohol-based sanitizers [17], as well as the high cost of these products [18], have been identified as barriers to their adoption.

Previous studies found that, factors such as educational level of the household head [11], media exposure [11] and household income status [11, 19], access to water and sanitation [11], household size [19] were determinants for both presence and absence of handwashing facility. To the best of our knowledge, only two previous studies on handwashing facility have been done in Sub-Saharan Africa. The first study looked at differences in access to water and soap in relation to wealth index and residence; while the second utilized a linear regression model to find variables of basic handwashing facility. Therefore, the study aims to determine the magnitude of limited handwashing facility and identify its determinants using a relatively efficient model. The study's findings will assist policymakers in many countries in establishing basic handwashing facility programs, which will help to reduce the spread of public infectious diseases and support future research.

## Methods

### Study setting and period

The Demographic and Health Surveys (DHS) program began in 1984 [20]. It is a nationally representative cross-sectional household survey conducted in low- and middle-income countries. We have used Demographic and Health Surveys (DHS) data which was conducted from 1st January 2010 to 31st December 2016 in 29 sub-Saharan African countries [21]. The survey is designed to collect information about maternal and child health, nutrition, household characteristics and other health issues. For comparison, the DHS survey adheres to the same basic protocols throughout the country. Households in DHS are selected using a two-stage cluster sampling methodology. In the first stage, cluster enumeration areas (EAs) (typically villages in rural areas or blocks in urban areas) were sampled using a probability proportional to the population size technique. In the second stage, all households in the selected area were listed, and then 25–30 households were chosen at random for interviews. This sampling strategy was utilized to get a representative sample of households. A total of 237,983 weighted samples were included in the study. All households located in sub-Saharan African countries were the source of population, while households found in 29 sub-Saharan African countries at the time of the DHS survey were the study population.

### Dependent variable

The outcome of this study was a limited handwashing facility. DHS collected information on handwashing facility, such as the place where handwashing facility found, whether fixed (such as Sink with tap and Tube with outlets) or mobile (such as Tippy tap, Raised bucket with tap/ outlet, Two buckets suspended, Suspended bottle or bag with outlet/hole/ pop-up plug and Foot pump sink). Furthermore, data on the presence of water, soap, and any detergents (ash, mud, or sand) on the premises were gathered through face-to-face interviews and observation. Based on this information, households with both fixed and mobile places and household members washing their hands without water and or soap (confirmed by observation) at the time of the interview, were considered as “having limited handwashing facility” [22].

### Independent variables

Individual and household level variables for limited handwashing facility extraction included the following; age of household head, sex of household head, marital status of the household head, household size, household wealth index, educational status of household head, floor material type (standard vs. substandard) [11], place of handwashing facility (fixed vs. mobile), water sources, sanitation facility and the number of under-five children in the household. Community-level factors that affected the availability of limited handwashing facility were place of residence, region, community-level education, income level, and community media exposure. Some of the individual variables were taken directly from DHS such as the sex of the household head. Other variables were computed and categorized further. The operational definition and coding of variables are summarized in the supplementary table (S 1& S 2).

### Data analysis

We have used STATA version 14.0 software to extract and analyze the data. After the samples were weighted, descriptive statistics were performed. Because of the hierarchical and clustering nature of the DHS data, a mixed effect multilevel logistic model was used. A cluster-level random intercept was utilized to determine the difference in limited handwashing facility between clusters. Meta-analysis was conducted to determine the pooled prevalence of limited handwashing facility in sub-Saharan Africa, as well as sub-group analysis by region, income level, and year of the survey were also employed.

Four models were fitted in the multilevel analysis. The first was a null model (Model 1) that was designed to check the variability in limited handwashing facility and which only contains the outcome variable. Model 2 and Model 3 were for individual/household and community-level variables, respectively. In the fourth model (Model 4), both the community and individual/ household variables were fitted simultaneously. Model comparison was done using deviance and the model with the lowest deviance was chosen as the best-fitting model.

### Ethical approval

Permission for data was obtained from the DHS program (https://dhsprogram.com/data/available-datasets.cfm). On the website, a request was made. The researchers had no ethical concerns since the DHS program handled ethical issues both before and throughout the survey**.**

## Results

### Indvidual and household level characterstices

For analysis, a total of 237,983 weighted samples were used. The average age of the household was 45 years. Most of the household heads were found between the ages of 35 and 60 years. However, when compared to other age groups, the highest limited handwashing facility (70.65%) was found over the age of 60 years. Overall, about 72.86% of the households were headed by men, and among them, 68.95% of household heads had limited handwashing facility. Around thirty-two percent (31.63%) of household heads had no formal education, and 82.46% of those household heads had limited handwashing facility. Less than one-fourth of households had a family size of seven or more, and 72.89% of those households had limited handwashing facility.

Among a respondents, 75.76% of households had improved water sources and 56.78% of households had improved sanitation facility. Three-fourths (76.32%) of the households had a mobile type of handwashing facility. Around twenty-seven (26.81%) of households spent more than 30 min in collecting water, of which 75.15% of households had a limited handwashing facility. The results of individual and household factors of limited handwashing facility are summarized in the table below (Table 1).

**Table 1:** Indvidual and household level factors of limited handwashing facility in sub-Saharan Africa, 2021: Data from the sub-Saharan afica Demographic and Health Survey since 2010 (*n* = 237,983)

Variables	Categories	Having limited handwashing facility		Total weighted frequency (%)
		**Yes 162,872 (68.44%)**	**No 75,111 (31.56%)**	
**Age of HH head**	**< 35**	55,874 (69.64%)	24,358 (30.36%)	80,231(33.71%)
	**35 – 60**	77,249 (66.80%)	38,397 (33.20%)	115,646 (48.59%)
	**> 60**	29,749 (70.65%)	12,356 (29.35%)	42,105 (17.69%)
**Sex of the head**	**Male**	119,558 (68.95%)	53,836 (31.05%)	173,394 (72.86%)
	**Female**	43,315 (67.06%)	21,274 (32.94%)	64,589 (27.14%)
**Educational status of HH head**	**No formal eduaction**	62,044 (82.46%)	13,198 (17.54%)	75,242 (31.63%)
	**Primary school**	54,028 (71.89%)	21,123 (28.11%)	75,151(31.59%)
	**Secondary school**	37,269 (58.51%)	26,432 (41.49%)	63,701(26.78%)
	**Higher**	9,471 (39.84%)	14,300 (60.16%)	23,771.47 (9.99%)
**Marital status of the head**	**Never married**	15,230 (56.57%)	11,694 (43.43%)	26,924 (11.31%)
	**Married**	118,005 (69.73%)	51,220 (30.27%)	169,225 (71.11%)
	**Widowed/divorced/separated**	29,638 (70.84%)	12,197 (29.16%)	41,835 (17.58%)
**Household size**	**1 -3**	55,103 (65.34%)	29,225 (34.66%)	84,328 (35.43%)
	**4 -7**	81,086 ( 69.28%)	35,963 (30.72%)	117,049 (49.18%)
	**7**^**+**^	26,683 (72.89%)	9,923 (27.11%)	36,606 (15.35%)
**Wealth index**	**Poor**	68,203 (82.26%)	14,713 (17.74%)	82,916 (34.84%)
	**Middile**	34,392 (75.17%)	11,359 ( 24.83%)	45,751 (19.22%)
	**Riche**	60,277 (55.14%)	49,039 (44.86%)	109,316 (45.93%)
**Handwashing facility**	**Fixed**	57,248 (57.49%)	42,334 (42.51%)	99,582 (41.84%)
	**Mobile**	105,624 (76.32%)	32,777 (23.68%)	138,401(51.16%)
**Source of water**	**Improved**	120,669 (66.93%)	59,626 (33.07%)	180,295 (75.76%)
	**Unimproved**	42,203 (73.16%)	15,485 (26.84%)	57,688 (24.24%)
**Sanitaion facilitiy**	**Improved**	82,859 (61.32%)	52,272 (38.68%)	135,131(56.78%)
	**Unimproved**	80,013 (77.79%)	22,839 (22.21%)	102,852 (43.22%)
**Time taken to get water**	**Less than 30**	113,199 (64.99%)	60,981 (35.01%)	174,180 (73.19%)
	**Above 30**	49,673 (77.85%)	14,130 (22.15%)	63,803 (26.81%)
**Housing/ floor material**	**Sutandared**	73,390 (58.19%)	52,739 (41.81%)	126,129 (53.00%)
	**Substandared**	111,854 (80.00%)	22,372 (20.00%)	111,854 (47.00%)
**Number of ** **under five**	**No chilidren**	69,528 (64.60%)	38,103 (35.40%)	107,631(45.23%)
	**1–2**	80,122 (70.97%)	32,768 (29.03%)	112,890 (47.44%)
	**3**^**+**^	13,222 (75.72%)	4,239 (24.28%)	17,461 (7.34%)

### Community level factors of handwashing faclitiy in SSA

About 95,549 (40.15%) of respondents live in urban areas. The community level education was the aggregate data from the educational status of the individual household head, and in this study, communties with low educational status accounted for 49.03%. Approximately half of the communities had high media exposure (50.80%) (Table 2).

**Table 2:** Community level factors of limited handwashing facility in sub-Saharan Africa, 2021: Data from the sub-Saharan Afica Demographic and Health Survey since 2010 (*n* = 237,983)

Varibles	Categories	Total wighted frequencey
Resdence	Urban	95,549 (40.15%)
	Rural	142,434 (59.85%)
Communtiy level education	Low	116,673 (49.03%)
	High	121,310 (50.97%)
Community level media exposure	Low	117,081 (49.20%)
	High	120,902 (50.80%)
Region	Esat Africa	105,346 (44.27%)
	Central Africa	22,299 (9.37%)
	West Afriaca	91,847 (38.59%)
	Southern Africa	18,491 (7.77%)
Income level	Low income	145,572 (61.17%)
	Lower middle	68,321 (28.71%)
	Upper middle	24,090 (10.12%)

### Prevalence of limited handwashing facility in sub-Saharan Africa

**Fig. 1:**
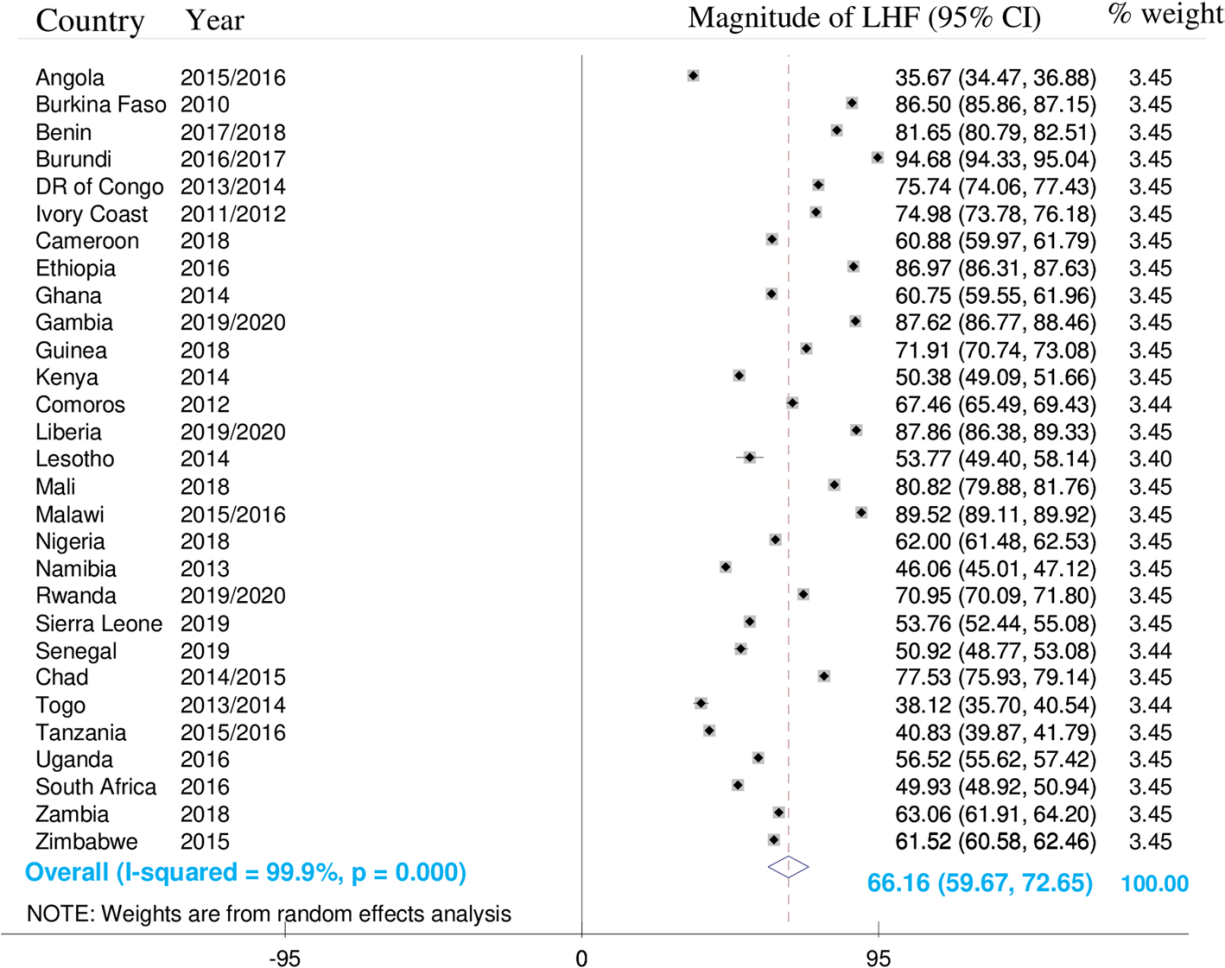
Pooled prevalence of handwashing facility among households in sub-Saharan Africa

In this study, the pooled prevalence of limited handwashing facility (households that lacked water and/or soap) was found to be 66.16% (95% CI; 59.67%—72.65%). I square (I ^2^) was 99.9%, which indicates that results for individual countries might vary to a larger extent from the pooled estimate. The pooled prevalence of having limited handwashing facility is summarized in the figure below “Fig. 1”.

### Sub-group analysis of limited handwashing facility by region

**Fig. 2:**
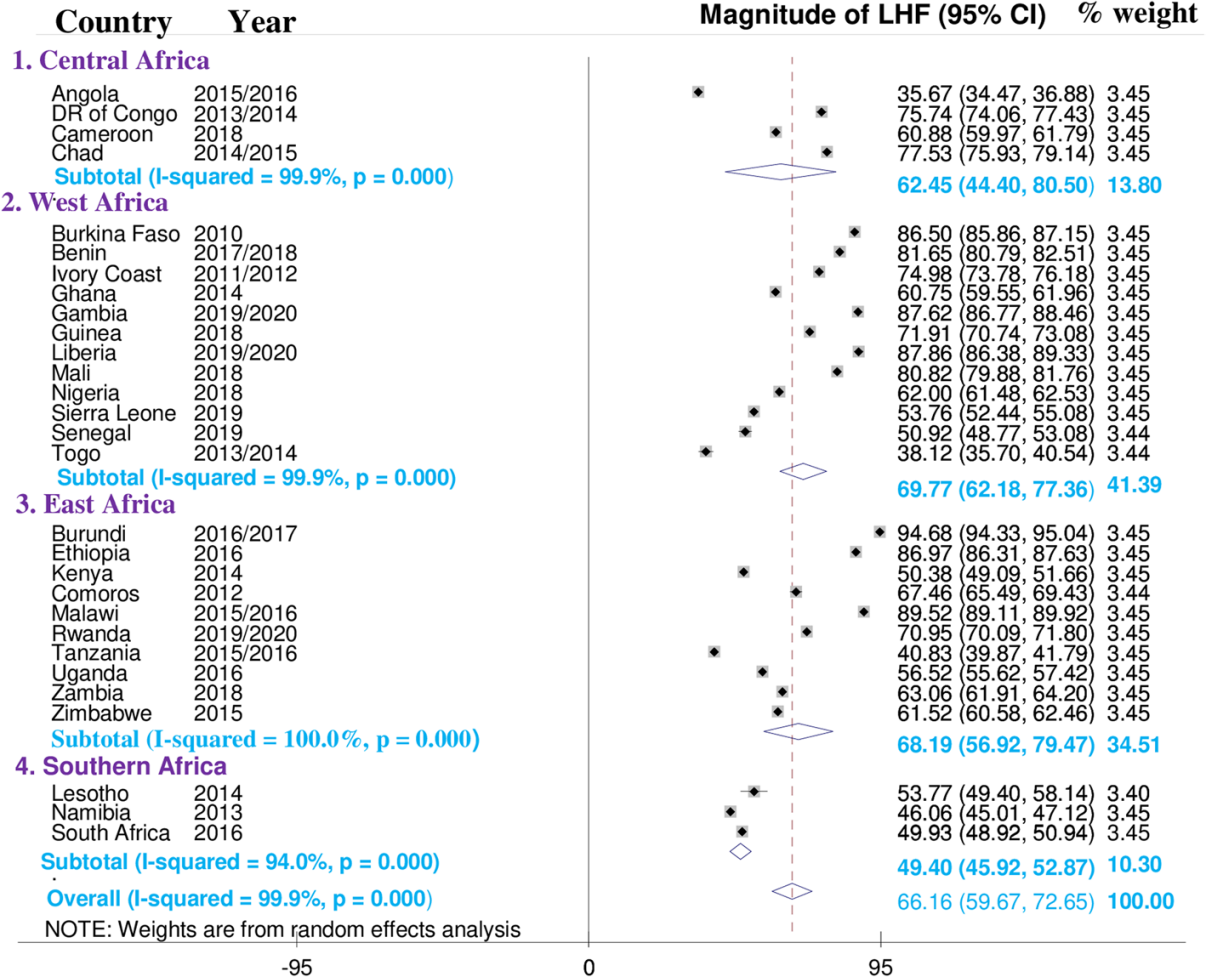
Sub-group analysis of limited handwashing facility by region in sub-Saharan Africa

I square result of this finding was high, indicating the presence of variability in pooled estimates across countries. To address this heterogeneity, a sub-group analysis was performed by region. Based on this, the magnitude of limited handwashing facility ranged from 49.40% (95% CI; 45.92—62.87) in southern Africa to 69.77% (95% CI; 62.18—77.36) in West Africa. The Central African region has fewer countries and a wider confidence interval. The following figure below summarizes the sub-group analysis of limited handwashing facility in region **“**Fig. 2”.

### Sub-group analysis of limited handwashing facility by income level of countries

**Fig. 3:**
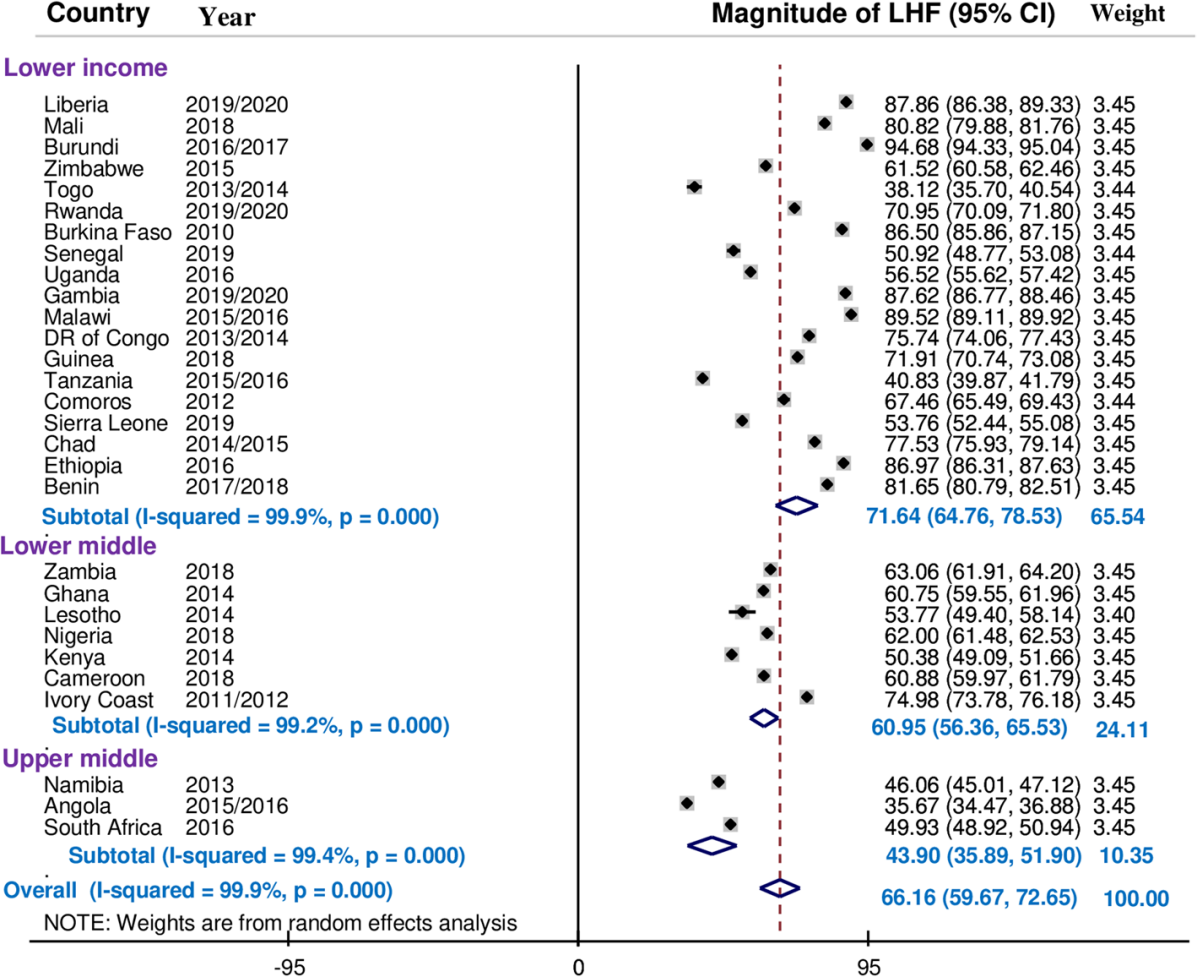
Sub-group analysis of handwashing facility among households in countries income status

We also conducted a sub-group analysis based on countries income level. According to the findings, the prevalence of limited handwashing facility was 71.64% (95% CI; 64.76—78.53) in low-income countries, 60.95% (95% CI; 56.36—65.53) in lower middle-income countries, and 43.90% (95% CI; 35.89—51.90) in upper-middle countries. The findings revealed that the prevalence of limited handwashing facility decreases as a country's income level rises. The figure below shows that a sub-group analysis based on countries income level “Fig. 3”.

### Sub-group analysis of limited handwashing facility by survey year

**Fig. 4:**
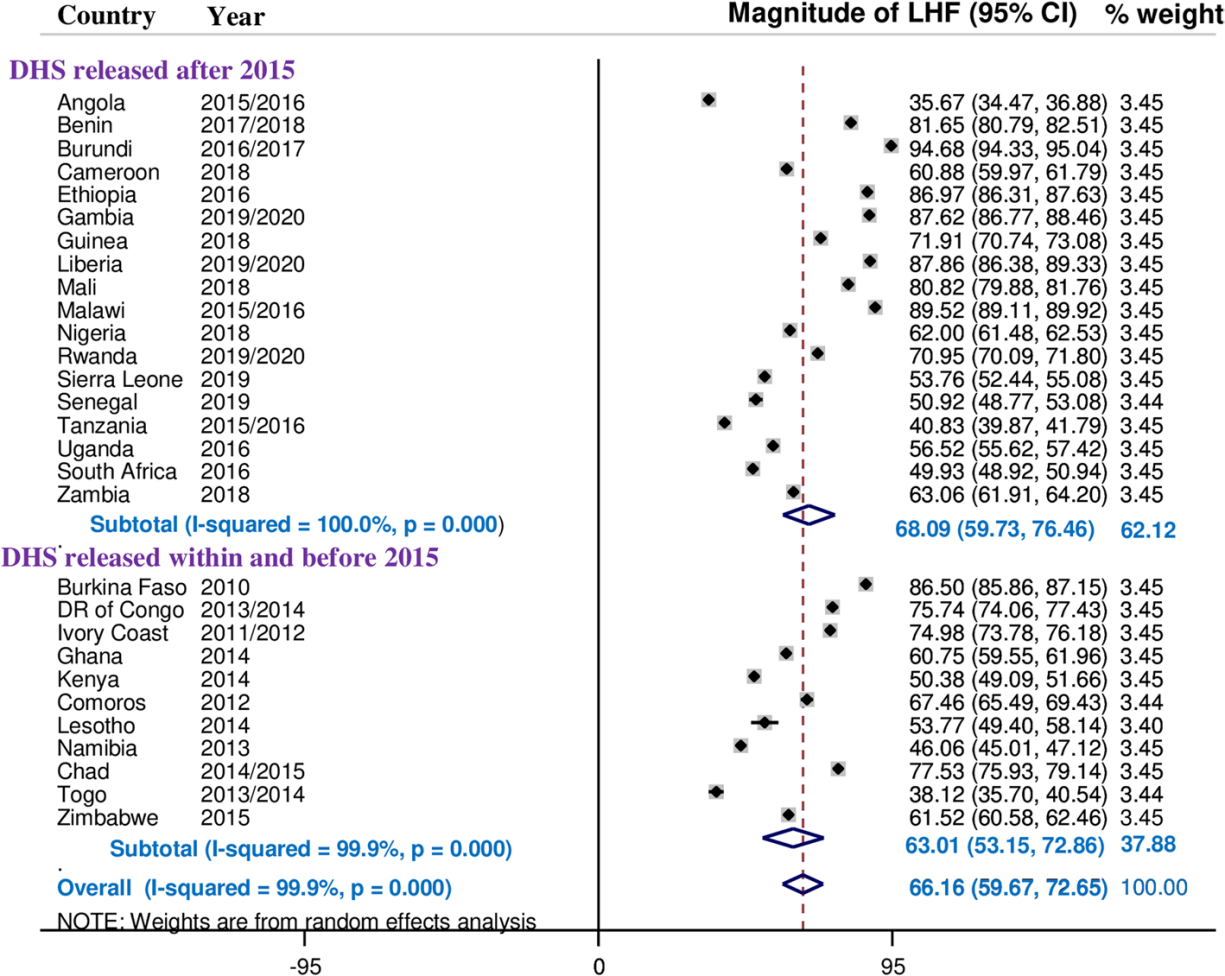
Sub-group analysis of limited handwashing facility in sub-Saharan Africa by survey year

We divided the survey year into two categories: above 2015 and below 2015. The prevalence of limited handwashing facility was 68.09% (95% CI; 59.73—76.48) among countries whose DHS survey was conducted above 2015 and 63.01% (95% CI; 53.15—72.86) among countries whose DHS survey was conducted below 2015. Burundi had the highest prevalence of limited handwashing facility at 94.68% (95% CI; 94.33—95.04) in the year 2016/2017, whereas Angola had the lowest prevalence at 35.67% (95% CI; 34.47—36.88) in the year 2015/2018. The sub-group analysis of limited handwashing facility by survey year is summarized in the figure below “Fig**. ****4**”**.**

### Random-effect and model comparison

As shown in Table 2, the Intraclass Correlation Coefficient (ICC) in the null model was 0.28, indicating that about 28% of the variations in limited handwashing facility between households were attributable to cluster differences, while the remaining 72% were assigned to individual household factors. The median odds ratio (MOR) between having the lowest and highest limited handwashing facility in the clusters was 2.93. Furthermore, the proportional change in variance (PCV) in the final model was 18.75%, indicating that the variation in limited handwashing facility among study households was explained by factors at both the individual and community levels simultaneously. The deviation test was used to compare and fit the models; the fourth model had the lowest deviation (259,616) and was chosen as the best-matched model [Table 3].

**Table 3:** Multilevel regression for factors of limited handwashing facility in sub-Saharan Africa

Variables	Categories	Null model	Model 1		Model 2	Model 3
			**AOR [95% CI]**		**AOR [95% CI]**	**AOR [95% CI]**
Age of HH head	< 35		0.94[ 0.91- 0.97]***			1.00 [0.96—1.02]
	35 – 60		0.83 [0.81- 0.86]***			0.89 [0.86—0.91]***
	> 60		Ref			Ref
Sex of HH head	Male		1.06 [1.04—1.08]***			0.99 [0.97—1.02]
	Female		Ref			Ref
Household size	1–3		0.90 [0.87—0.93]***			0.94 [0.91—0.98]
	4–7		0.92 [0.89—0.95]***			0.91 [0.88—0.94]
	7^+^		Ref			Ref
Place of handwashing facility	Fixed		Ref			Ref
	Mobile		2.22 [2.18—2.26]***			1.73 [1.70—1.77]***
Source of water	Improved		Ref			Ref
	Unimproved		1.09 [1.06—1.11]***			0.98 [0.96—1.01]
Sanitation facility	Improved		Ref			Ref
	Unimproved		1.89 [1.86—1.94]***			1.58 [1.55—1.62]***
Time taken to get water	< 30 min		Ref			Ref
	> 30 min		1.45 [1.41—1.48]***			1.16 [1.13—1.19]***
Children 5 years and below	No children		Ref			Ref
	1–2		1.17 [1.14—1.20]***			1.11 [1.09—1.14]***
	3^+^		1.33 [1.27—1.39]***			1.25 [1.20—1.31]***
Residence	Rural				2.71[ 2.66—2.77]***	2.08 [2.04—2.13]***
	Urban				Ref	Ref
Com. Media	Low				1.50 [1.32—1.70]***	1.47 [1.31—1.66]***
	High				Ref	Ref
Income level	Low income				3.11[3.02—3.21]***	2.41 [2.33—2.49]***
	Lower middle				2.43[ 2.35—2.51]***	2.10 [2.14—2.17]***
	Upper middle				Ref	Ref
Com. Education	Low				1.30 [1.13—1. 48]***	1.30 [1.14—1.48]***
	High				Ref	Ref
**Random effects**						
	**VA**	1.28	1.11	1.11		1.01
	**ICC**	0.28	0.25	0.25		0.23
	**MOR**	2.93	2.72	2.72		2.60
	**PCV**	Reff	13.28	13.28		21.09
**Model comparison**						
	**Deviance**	283,410	267,606		264,968	259,616

*ICC* Intraclass correlation coefficient, *MOR* Median odds ratio, *PCV* Proportional change in variance, Com. Media = Community media use; Com. Education = Community educational status

^*^P-value < 0.05

^**^Pvalue < 0.01

^***^Pvalue < 0.001

### Mixed-effect analysis of factors associated with limited handwashing facility

This study covered factors at the individual, household, and community levels. Variables with p values less than 0.25 were eligible for further multilevel analysis. Individual and household variables such as the age of the household head, the sex of the household head, household size, location of handwashing facility, source of water, sanitation facility, time taken to get water, and the number of under-five children were selected while community-level factors such as residence, community media exposure, income level and community level education were selected for further analysis.

In the final model (model 4), age of the household head, place of handwashing facility, sanitation facility, time taken to get water, number of under-five children, residence, media exposure, income status and level of education were all associated with limited handwashing facility.

Household heads aged between thirty-five and sixty had 11% of limited handwashing facility with [AOR = 0.89, 95% CI; 0.86—0.91]. Households with a mobile type of handwashing facility were 1.73 [AOR = 1.73, 95% CI; 1.70—1.77] times more likely to have limited handwashing facility than those with a fixed location.

In terms of sanitation, households with unimproved sanitation were 1.58 [AOR = 1.58, 95% CI, 1.55, and 1.62] times more likely to have limited handwashing facility than those with improved sanitation. The odds of having a limited handwashing facility were 1.16 [AOR = 1.16, 95% CI; 1.13–1.19] times higher in households that had to travel more than 30 min to get water than in households that got water within 30 min. When we compared urban household residents, the odds of having a limited handwashing facility in rural areas was 2 times higher [AOR = 2.08, 95% CI; 2.04—2.13]. Communities with low media exposure had 1.47 [AOR = 1.47, 95% CI; 1.31—1.66] times higher odds of having limited handwashing facility than those communities with higher media exposure. The likelihood of having a limited handwashing facility was 1.3 [AOR = 1.30, 95% CI; 1.14—1.48] times higher in low educated communities than in higher educated communities. The likelihood of having a limited handwashing facility was 2.41 [AOR = 2.41, 95% CI; 2.33—2.49] and 2.1 [AOR = 2.10, 95% CI; 2.14—2.17] times higher among low-income and lower-middle-income countries as compared with upper-middle countries, respectively.

The odds of having limited handwashing facility was 1.11 [AOR = 1.11, 95% CI; 1.09—1.14] and 1.25 [AOR = 1.25, 95% CI; 1.20—1.31] times higher in households with at least two and three or more children, respectively, than in households without children [Table 3].

## Discussion

The pooled prevalence of limited handwashing facility (lack of water and/or soap) in this study was 66.16%, with a 95% confidence interval of (59.67%—72.65%).This result was higher than that of a surveys conducted in 44 countries [9] and Ethiopia [11], where the population living in households with a limited handwashing facility was 35.5 ± 23.7% and 54.04% respectively. The discrepancy in magnitude from 44 countries might be due to the differences in socio-economic characteristics of countries. In our case, the majority of countries (61.17%) were classified as low income according to the World Bank classification, 2019. In the case of Ethiopia, there was a small sample size and there might be the difference in fixation of the outcome variable. However, the current finding was lower than the Joint monitoring program (WHO & UNICEF) report in 2017, which found that 85% of sub-Saharan Africans have limited handwashing facility [10]. The explanation for this could be that the report includes all SSA countries, but in our case, we only included 29 countries. Besides, the households might not change in awareness and income status to basic handwashing facility within the year range.

Individual, household, and community-level factors for handwashing facility were all included in this study. Based on multilevel regression model, household heads with older age was significant and a protective factor of limited handwashing facility. The finding is supported by different studies [23–25], where older household heads had low limited handwashing facility than younger household heads. This could be because older people's immune systems deteriorate with age, and they may require care to avoid infection. The age difference in this study highlights the necessity for the government to establish and implement age-specific educational programs or campaigns.

According to the current study, having low media exposure in the community had higher effect on the presence of limited handwashing facility. The result was supported by different studies [11, 24], where having limited handwashing facility were increased with no having radio and television. Radio and television are powerful and efficient ways to conduct coordinated national awareness campaigns [11]. These devices can send messages to a huge number of people at the same time. Radio is highly vital for reaching out to handwashing messages, especially in remote rural areas where there is no electrical supply. Television is also very important for handwashing demonstration techniques. Handwashing campaigns through media results in lower health consequences for the community [24]. This finding revealed that each county's administration may improve handwashing facility by expanding mass media coverage in both rural and urban areas.

Countries with low and lower middle-income levels were associated with the presence of limited handwashing facilities; this finding was supported by different studies [19, 26–29], which found that the richest households were more likely to have effective handwashing facility than the poorest households. However, this finding was inconsistent with studies done in developing countries [23, 30], where countries with higher Gross Domestic Product have limited handwashing facility. The current study is interesting in that those countries with lower financial status cannot afford to pay for water services, and purchasing soap/detergents may be crucial factor. The government can address this issue by providing targeted sanitation and hygiene subsidies in the community.

Households who travel more than 30 min round trip to get their water source had a limited handwashing facility. This finding was reinforced by research in Kenya [28]. But it contradicted another study [23], which found that households having a water supply in their dwelling were less likely to have a handwashing facility. The reason for the disparity could be that households are less motivated to use water for handwashing and do not perceive themselves to be susceptible to infection [23]. However, when water is brought from a distance, the person's handwashing activities are less likely to occur at a vital period. If people acquire their water from a remote location, people may prefer to save water for drinking rather than washing hands. The findings indicated that handwashing facility may be enhanced if the government provided an accessible water supply system.

Our study also found that communities with a low educational level were more likely to have limited handwashing facility. This finding is supported by evidence that those who received basic handwashing education were more likely to have handwashing facility [1]. It was also supported by the previous studies [23, 26, 28], where having effective handwashing facility were gradually increased with the increment of the education level of the head of the household. Health education is critical in raising information about the ability to wash hands with soap since uneducated household heads did not know the facilities needed for proper handwashing [31]. Lack of hand hygiene knowledge with other factors such as limited resources of a household may have a positive effect on the presence of limited handwashing facility particularly in low- income countries. However, evidence suggests that health education program on handwashing are more important than investing in handwashing facilities [32]. To improve handwashing facility, the government should increase community awareness and behavior through mass media campaigns and community-level education.

According to our findings, households with more children have limited handwashing facility. This is a contradictory pattern from East African countries [23], where handwashing facility is less prevalent in households without children. The possible explanation in our situation might be that a household with many children is costly to have a basic handwashing facility, and children lack proper use of the available handwashing facility. Having a large number of children, on the other hand, could be a big opportunity for future home hygiene practice since they are more sensitive to learning and are very likely to adopt healthy behaviors at a younger age [33].

Residence was also another factor for the limited handwashing facility. In this study, being rural residents were more likely to have limited handwashing facility than urban areas. The finding was supported by other evidences [23, 24, 27]. Challenges in rural locations related to the cost of obtaining soap or clean water may be the reason for the presence of limited handwashing facility [23, 26]. The reason might be that the rural individuals lacked the necessary education and information on risk of infection related to having limited handwashing facility. Thus, improving educational levels of rural households will close the gap between urban and rural areas. Moreover, the government can improve handwashing facilities by implementing a handwashing health service package program.

Unimproved sanitation facilities were associated with limited handwashing facility. The finding was supported by suggested evidences [26, 28], households with a shared type of sanitation facility were less likely to have an effective handwashing facility. Unimproved sanitation and unprotected water sources are also indicated as having limited handwashing facility [26]. One possible justification for this is that poor designed sanitation facilities in household may lacked water and soap on-premises to wash hands after toilet visit. The like hood of having a limited handwashing facility was higher in a mobile type of handwashing facility. This was supported by another study, which found a higher prevalence of handwashing with soap in areas having a handwashing station within 10 paces of the kitchen [19]. The finding is expected that utilizing soap and water for handwashing is inconvenient unless there is a fixed location. The government should demonstrate how to install improved sanitation with handwashing facility using locally available materials.

### Implication of the study

This study's findings are critical because it will assist the government and WASH stakeholders in improving the educational status of households and determining the best alternative choices for the affordability problem, which mostly affects handwashing practices. Work on handwashing minimizes costs associated with the treatment of infectious diseases, including the present COVID-19 pandemic, because prevention is better than cure. The COVID-19 pandemic is a severe disease, and handwashing with soap and water, in conjunction with other measures, will be a useful strategy for avoiding further COVID-19 transmission. Since good handwashing is extremely difficult in the absence of adequate handwashing facility, the pandemic disease will persist.

### Limitation

We used DHS (secondary) data, which is cross-sectional in nature and reflects the handwashing status that exists only at the time of the survey. DHS does not collect all of the variables that must be considered when it comes to handwashing facility (e.g., social, cultural, behavioral). As a result, despite our findings expressing existing evidence, other confounding factors may exist. We were only interested in the presence or absence of a handwashing facility, not handwashing practice, so having limited handwashing may not be considered poor handwashing practice.

## Conclusion

In this study, the pooled prevalence of limited handwashing facility (without water and or soap) is 66.16%. West African region had the highest prevalence of limited handwashing facility when compared to others regions in sub-Saharan Africa. The prevalence of limited handwashing facility increase when income level reduced. The age of the household head, location of the handwashing facility, number of under-five children, household size, sanitation facility, residence, income level, community-level media exposure, and education were a determinant factors for limited handwashing facility.

### Recommendation

Organizations and government agencies of low and low-middle-income countries should better to build handwashing water infrastructure and providing or supplying soap with low cost for households that cannot buy soap. The Global Handwashing Partnership and other respected health sectors in each country should promote handwashing for rural residents, for whom washing hands with soap may be considered luxurious due to poor educational levels. Furthermore enhancing the knowledge of risks related to poor handwashing practice is very important. For researchers, focusing on personal behaviors as well as societal and cultural determinants of limited handwashing facility through qualitative research will be more informative. Finally, governments in each country should prioritize handwashing programs based on the observed factors.

## Supplementary Information

**Additional file 1: Supplementary table (S1).** Individual level variables extracted from DHS data set for studying factors associated with limited handwashing facility. **Supplementary table 2 (S2).** Community level variables extracted from Demographic and Health Survey data set for studying factors associated with limited handwashing facility.

## Data Availability

Data is available publically access from the open databases. It can be accessed by the following website.https://dhsprogram.com/data/dataset_admin/login_main.cfm?CFID=10818526&CFTOKEN=c131014a480fe56-4E0C6B7F-F551-E6B2-50.

## Abbreviations

AOR: Adjusted odds ratio
CI: Confidence interval
HH: Household
DHS: Demographic and health survey
LHF: Limited handwashing facility
SSA: Sub-Saharan Africa
UNICEF: United Nations children's fund
WHO: World health organization
