# Supplementary material for: Limited handwashing facility and associated factors in sub-Saharan Africa: pooled prevalence and multilevel analysis of 29 sub-Saharan Africa countries from demographic health survey data
Source: BMC Public Health. 2022 Oct 27;22:1969. doi: 10.1186/s12889-022-14390-4 (PMC9610344; doi:10.1186/s12889-022-14390-4)
Supplement: Supplementary file 1 — Additional file 1: Supplementary table (S1). Individual level variables extracted from DHS data set for studying factors associated with limited handwashing facility. Supplementary table 2 (S2). Community level variables extracted from Demographic and Health Survey data set for studying factors associated with limited handwashing facility. [file 12889_2022_14390_MOESM1_ESM.docx]

**Supplementary table (S1): Individual level variables extracted from DHS data set for studying factors associated with limited handwashing facility.**

| Variable | Definition | | Categories | |
| --- | --- | --- | --- | --- |
| Age | The age of the household head in years at the time of the survey | | 0. < 35 years  1. 35 - 60 years  2. > 60 years | |
| Sex | The sex of the household head | | 1. Male  2. Female | |
| Household head Education | The highest educational level the household head attained at the time of survey | | 0. No formal education  1. Primary  2. Secondary  3. Higher | |
| Marital status | Marital status of the household head at a time of survey | | 0. Never married  1. Married  2. Windowed/Divorced/separated | |
| **Household variables extracted from DHS** | | | | |
| Wealth index | | Categories were given based on the number and kinds of consumer goods the households owned | | 0. Poor  1. Middle  2. Rich |
| Household size | | The number of household members with which the household head was living. | | 0. < 3  1. 4-7  2. 7^+^ |
| Sanitation facility | | **Improved**: flush - flush - to septic tank, flush - to pit latrine, flush – unspecified, pit latrine - ventilated improved pit (vip), pit latrine - with slab and composting toilet  **Unimproved:** flush - to somewhere else, flush - don't know where, pit latrine - without slab / open pit, no facility/bush/field, bucket toilet, hanging toilet/latrine and other | | 0. Improved  1.Unimproved |
| Source of drinking water | | **Improved**: When water source is: piped water (piped into dwelling, piped to yard/plot, piped to neighbour), public tap/standpipe, tube well, or borehole  **Unimproved**: When water source is: dug well (open/protected), protected well,  unprotected well, surface (spring, river, dam, lake, ponds, stream, canal or irrigation  channel), protected spring, unprotected spring, rainwater, or tanker truck | | 0. Improved  1. Unimproved |
| Location of handwashing facility | | Place of handwashing facility the household members used to wash their hands | | 0. Fixed  1. Mobile |
| Housing condition  (floor) | | **Standard**: finished, parquet or polished wood, vinyl or asphalt strips, ceramic tiles, cement, or carpet  **Sub-standard**: natural, earth/sand, dung, rudimentary, wood planks and palm/bamboo | | 0. Standard house  1.Substandard house |
| Time to get water | | Water available on premises plus accessed in less than 30 minutes in round trip vs. requires greater than 30 minutes to access | | 0. < 30 minutes  1. > 30 minutes |
| Number of children under five | | Number of under-five aged children in the household at the time of survey | | 0. No child  1. 1 to 2  2. 3^+^ |

**Supplementary table 2 (S2) : Community level variables extracted from Demographic and Health Survey data set for studying factors associated with limited handwashing facility**

| Variables | Description | Category |
| --- | --- | --- |
| Place of residence | The place where the household head residing at a time of survey | 0. Urban  1. Rural |
| Countries income level | According to World Bank 2019, countries were classified based their Gross National Income (GNI) status. | 0. Low income  1. Lower middle  2. Upper middle |
| Mass media exposure | Defined as the proportion of household heads who  had mass media exposure within the cluster. The aggregate of individual household heads with mass media exposure can show overall mass media exposure of the cluster. It was categorized as high if cluster has more than 50% of household heads with mass media exposure or low otherwise | 0. Low  1. High |
| Community Educational status | Defined as the proportion of household head who  attended primary/secondary/higher education within the cluster. The aggregate of individual household head's primary/secondary/higher educational level can show overall educational attainment of the household head in the cluster. It was categorized as high if clusters with more than 50% of primary/secondary/higher education or low otherwise. | 0. Low  1. High |
| Region | The sub region category of sub-Saharan African countries was based on sustainable development goal classifications. | 1. East Africa  2. Central Africa  3. West Africa  4. Southern Africa |
